# Potential clinical application of an automated fluorescent microbial cell counter in the detection of urinary tract infection

**DOI:** 10.1002/jcla.23334

**Published:** 2020-07-04

**Authors:** Laila E. Phillips, Sandeep Verma, Balarama K. Surapaneni, Sudhir K. Dutta

**Affiliations:** ^1^ Division of Gastroenterology Research Sinai Hospital Baltimore MD USA; ^2^ Department of Internal Medicine Aventura Hospital and Medical Center Aventura FL USA; ^3^ University of Maryland School of Medicine Baltimore MD USA

**Keywords:** cellular quantification, clinical diagnostics, *Escherichia coli*, microbiology, urinary tract infection

## Abstract

**Background:**

Urinary tract infections (UTI) account for millions of office visits and approximately 400 000 hospital admissions every year in the United States; as a result, the cost burden of UTI in the USA is estimated at approximately $2.8 billion. There is a great deal of interest in finding newer, faster, and more reliable methods for diagnosing UTI as compared to the standard urine culture.

**Methods:**

An automated fluorescent microbial cell counter was used to compare urine samples found to be positive for *Escherichia coli* UTI via cell culturing (n = 11) with UTI‐negative samples (n = 10).

**Results:**

Patients with a positive urine culture had significantly higher cell count results using the microbial cell counter (1.01 × 10^8^ cells/mL) as compared to the negative samples (2.35 × 10^6^ cells/mL; *P* = .0022).

**Conclusions:**

These observations suggest that automated microbial cell counters may serve as a rapid, objective method for the detection of bacteriuria in urine samples submitted for evaluation of suspected UTI.

## 1 INTRODUCTION

In 2011, urinary tract infections (UTI) accounted for around 400 000 hospitalizations, resulting in an estimated cost burden of approximately $2.8 billion in the United States.^1^ Fifty percent of women experience a UTI by age 35, and it has been estimated that over 7 million physician's office visits occur due to this common infectious disease.^1^, ^2^

The most common risk factors for UTI are a personal or familial history, frequent sexual intercourse, and spermicide use.^3^

A rapid urinalysis is usually conducted upon presentation with UTI‐related symptoms in patients. A rapid urinalysis screens the urine for ketones, proteins, reducing substances, red blood cells (RBC), white blood cells (WBC), nitrites, and pH levels outside the normal range (4.5 to 8.0).^4^ There are several methods used for preliminary screening for bacteriuria, or the presence of bacteria in the urine, which may be indicative of bacterial colonization of the urinary tract. These include measuring levels of leukocyte esterase in the urine, which is suggestive of leukocytosis in response to bacterial infection, a dipstick nitrite test that screens for urinary nitrites formed by bacterial oxidation, and microscopic quantification of microbial cells in a urine samples into “mild,” “moderate,” or “heavy” bacteriuria.^5^ The sample is ideally clean‐catch and mid‐stream, and collected in the morning. This reduces contamination of the tested sample by the normal urinary tract or fecal flora.

A full urinalysis involves analyzing urine upon an abnormal result on a rapid urinalysis test. A full urinalysis involves screening the urine for abnormal color, odor, specific gravity, and chemical composition via dip‐sticks and may involve a microscopic examination to identify bacteria, red blood cells, white blood cells, casts, and crystals.^5^ A urinalysis suggestive of bacteriuria is followed by a urine culture to determine the specific infecting organism. The most common pathogen associated with UTI globally is *Escherichia coli*.^6^, ^7^ The appropriate treatment is administered depending on the type of infection detected.

Catheterization studies intended to localize the site of bacteriuria demonstrated notable bacterial concentrations (300‐1000 bacteria/mL) in cytoscopic specimens collected following bladder irrigation from healthy subjects.^8^ This finding suggests the importance of quantifying bacteria in the urinary tract, especially where UTI is concerned.

There is a great deal of interest in finding newer, faster, and more reliable methods for diagnosing UTI. Automated cell counters that can provide a rapid and accurate quantification of cells of a particular size and volume in a fixed volume of specimen have opened new avenues for screening urine samples for the presence of pathogens in a diagnostic setting. This method of assessing bacterial concentration may reduce the need to perform culture‐sensitivity tests on samples that were suspected to be infected by the conventional urinalysis methods.

Automated fluorescent cell counters such as the Countess™ (Invitrogen/Thermo Fischer Scientific) have been used extensively for assays requiring cell quantification, protein expression, and cellular viability. Most sample preparations for automated cell counters involve staining a cellular suspension with a fluorescent nucleic acid‐binding dye, which is then analyzed by the machine. However, most automated cell counters have only been optimized for use in detecting cells within the 5‐10 μm range. Bacterial cells tend to be approximately ten‐fold smaller than plant and animal cells; on average, their diameters can range from 0.2 to 10 μm. *E. coli* cells are 1‐1.1 μm in diameter and 2 μm long.^9^

Prior versions of cell counters excluded many microbial species from their repertoire, but counters able to handle cells in the size range of 0.3‐50 μm have recently become available. In the present example, we tested a microbial cell counter that utilizes a nucleic acid‐staining dye able to permeate both Gram‐positive and Gram‐negative bacteria, thus providing a total cell count. Like in other cell counters, a cell suspension is stained with a fluorescent dye, and upon centrifugation, the suspended cells occupy a single focal plane. The cell counter quantifies the cells through automated fluorescence imaging and analysis based on an automated algorithm. This algorithm includes the ability to decluster stained objects to consider each tagged cell separately, providing an accurate cell count as compared to a subjective assessment of cells under the microscope.

The objective of this study was to determine the capacity for an automated microbial cell counter to detect microbial cells and robustly differentiate between urine samples previously identified as positive and negative for UTI via standard urinalysis culturing techniques. These results indicate a potential role for an automated microbial cell counter in a rapid clinical diagnostic setting.

## 2 MATERIALS AND METHODS

### 2.1 Study subjects

This research was conducted under a local IRB‐approved protocol and received Exempt status. Clean‐catch urine samples from healthy control subjects with a negative urinalysis result (n = 10) and subjects with suspected UTI with a positive urinalysis and culture result for *E. coli* (n = 11) were provided by the Microbiology Laboratory at Sinai Hospital of Baltimore. The culture result of each sample was not known to the technician prior to analysis. No demographic or personal information was provided with the samples.

### 2.2 Urine culture

Urine culture protocol was provided by the Microbiology Laboratory at Sinai Hospital of Baltimore (Maryland, USA). Urine samples that received a positive result were plated onto blood agar and MacConkey medium for growth analysis. This inoculation technique is known as direct surface plating, which is considered a standard quantitative culture method.^8^ Half of the plate contained blood agar, on which Gram‐positive and Gram‐negative bacteria can grow, whereas the other half contained a selective medium (in this case, MacConkey), on which Gram‐negative bacteria can grow while Gram‐positive growth is inhibited.^8^ A colony count was estimated following incubation and reported as cfu/mL of urine. Growth > 10^5^ cfu/mL was considered a positive result for UTI.

### 2.3 QUANTOM Tx™ urine sampling

Urine samples were analyzed using the QUANTOM Tx™ Microbial Cell Counter (Logos Biosystems, Anyang‐si, Gyeonggi‐do, South Korea) upon reception from the microbiology laboratory. All samples and reagents were vortexed thoroughly prior to use. For each sample, 1 µL QUANTOM™ Cell Staining Dye (Cat. # Q13101; Lot #QD0AGD2401) and 1 µL QUANTOM™ Total Cell Staining Enhancer (Cat. # Q13002; Lot # QE0AGD2401) was added to 10 µL of urine in a sterile microfuge tube. 8 µL of QUANTOM™ Cell Loading Buffer I (Cat. # Q13001; Lot # LB0AGC2101) was added and mixed by pipetting up and down. Once mixed, 6 µL of stained urine sample was added to a QUANTOM™ M50 Cell Counting slide chamber (Cat. # Q12001; Lot #12160501) in triplicate. Each slide was spun in the QUANTOM™ Centrifuge at 300× RCF for 10 minutes. Following centrifugation, each sample was read using default counting settings (20 autofocused images).

### 2.4 Statistical analysis

The statistical mean of the triplicated measurements for each sample was calculated. The statistical mean, range, and standard deviation for each group were then calculated using these values. Statistical significance (*P* < .5) was established using a heteroskedastic two‐sample *t* test.

## 3 RESULTS

**Figure 1:**
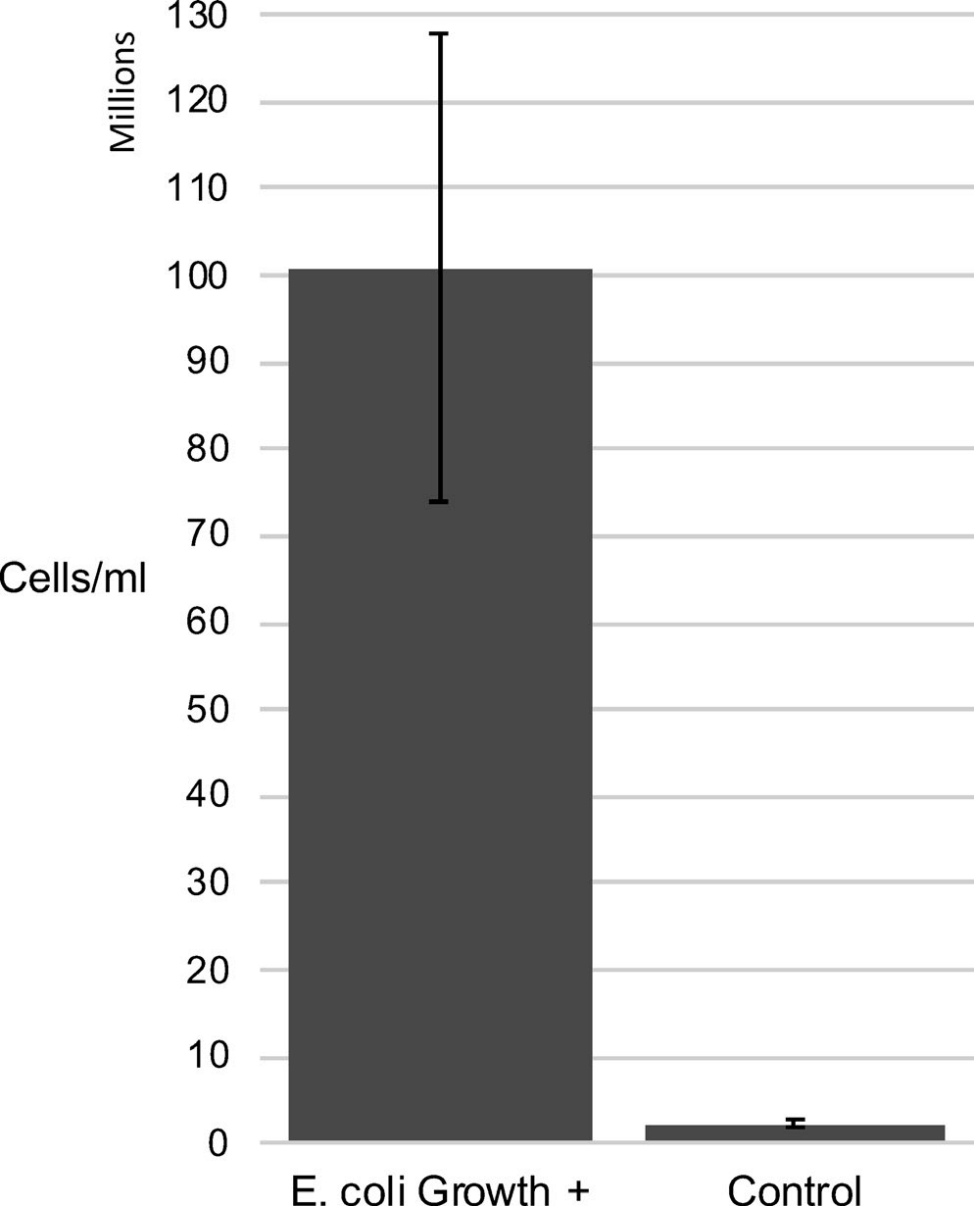
Bar graph demonstrating mean values of cells detected in units of millions of cells/mL for control samples (associated with clinically negative *Escherichia coli* growth) and clinically positive *E*. *coli* samples. The error bars represent the standard error of measurements for control samples (n = 10) and clinically positive *E coli* samples (n = 11)

The average cellular concentration for *E*.* coli*‐positive samples (n = 11) was 1.01 × 10^8^ cells/mL (range = 2.5 × 10^7^–3.29 × 10^8^; SD = 8.9 × 10^7^; Figure 1). The average cellular concentration for control samples (n = 10) was 2.35 × 10^6^ cells/mL (range = 9.42 × 10^5^–5.93 × 10^6^; SD = 1.56 × 10^6^; Figure 1). The difference in cellular concentration between the *E*.* coli*‐positive and control groups was found to be statistically significant via a heteroskedastic two‐sample *t* test (2.33 × 10^−2^ cells/mL mean difference, *P* = .0022; Figure 1).

## 4 DISCUSSION

The assessment of bacteriuria guides the treatment of suspected UTI based on clinical symptoms. Currently, bacteriuria is diagnosed indirectly by microscopic and chemical analysis of the urine. In the present study, the significant difference found between total cell counts in urinalysis‐negative and *E*. *coli*‐positive urine samples indicates that an automated microbial cell counter could provide a rapid, robust, and high‐throughput initial screening method for UTI. The use of an automated microbial cell counter could decrease the burden on microbiology laboratories and healthcare systems through efficient and accurate bacterial quantification. This technology could be particularly useful in clinical settings where rapid results are crucial and would expedite the overall management of patients with UTI in outpatient, ER, and express care unit settings.

Our findings may also suggest a potential for culture‐independent methods of assessing UTI. In the present study, the microbial cell counter was not used to identify bacterial species present in urine. An important feature of standard urine culture techniques is the ability to identify the type of bacteria present in the urine and its sensitivity to antimicrobials, which allows for more specific treatment to be administered depending on the kind of infection. However, with advances in nucleic acid‐binding fluorescent chemistry, cellular quantification technology, and software, the ability to rapidly identify bacterial species may soon become reality.

Additional prospective controlled clinical trials are needed to define the precise role of the present state of this technology in the management of patients with UTI or other types of infection. Such studies should include analysis of samples associated with asymptomatic bacteriuria (ASB) to determine the extent to which total bacterial counts differ between ASB and symptomatic UTI.

## 5 CONCLUSIONS

The automated microbial cell counter represents a significant step toward high throughput, reproducible microbial cell observation, and quantification. A significant difference in cellular concentration was observed between *E*. *coli*‐positive UTI samples and controls measured with an automated microbial cell counter. Thus, automated microbial cell counters may serve important roles as preliminary screening tools in clinical diagnostic settings.
